# 3-(5-Nitrofuran-2-yl)prop-2-en-1-one Derivatives, with Potent Antituberculosis Activity, Inhibit A Novel Therapeutic Target, Arylamine *N*-acetyltransferase, in Mycobacteria

**DOI:** 10.3390/antibiotics9070368

**Published:** 2020-07-01

**Authors:** Neha Agre, Nilesh Tawari, Arundhati Maitra, Antima Gupta, Tulika Munshi, Mariam Degani, Sanjib Bhakta

**Affiliations:** 1 Department of Pharmaceutical Sciences and Technology, Institute of Chemical Technology, Nathalal Parekh Marg, Matunga, Mumbai 400019, India; neha.agre@yahoo.com (N.A.); tawari.nilesh@gmail.com (N.T.); 2 Department of Biological Sciences, The Institute of Structural and Molecular Biology, Birkbeck, University of London, Malet Street, London WC1E 7HX, UK; amaitr01@bbk.ac.uk (A.M.); Antima.Gupta@lshtm.ac.uk (A.G.); 3 Faculty of Infectious and Tropical Diseases, London School of Hygiene & Tropical Medicine, Keppel Street, London WC1E 7HT, UK; 4 Institute for Infection and Immunity, St George’s, University of London, Cranmer Terrace, London SW17 0RE, UK; tmunshi@sgul.ac.uk

**Keywords:** tuberculosis, antibiotic resistance, 5-nitrofuran, arylamine *N*-acetyltransferase

## Abstract

In this study, the inhibitory potential of 3-(5-nitrofuran-2-yl)prop-2-en-1-one derivatives was evaluated against a panel of bacteria, as well as mammalian cell lines to determine their therapeutic index. In addition, we investigated the mechanism of antibiotic action of the derivatives to identify their therapeutic target. We discovered compound **2** to be an extremely potent inhibitor of *Mycobacterium tuberculosis* H37Rv growth (MIC: 0.031 mg/L) in vitro, performing better than the currently used first-line antituberculosis drugs such as isoniazid, rifampicin, ethambutol, and pretomanid in vitro. Furthermore, compound **3** was equipotent to pretomanid against a multidrug-resistant *M. tuberculosis* clinical isolate. The derivatives were selective and bactericidal towards slow-growing mycobacteria. They showed low cytotoxicity towards murine RAW 264.7 and human THP-1 cell lines, with high selectivity indices. Compound **1** effectively eliminated the intracellular mycobacteria in a mycobacteria-infected macrophage model. The derivatives were assessed for their potential to inhibit mycobacterial arylamine *N*-acetyltransferase (NAT) and were identified as good inhibitors of recombinant mycobacterial NAT, a novel target essential for the intracellular survival of *M. tuberculosis*. This study provided hits for designing new potent and selective antituberculosis leads, having mycobacterial NAT inhibition as their possible endogenous mechanisms of action.

## 1. Introduction

Tuberculosis (TB), caused by *Mycobacterium tuberculosis*, ranks above HIV/AIDS in mortality and is the leading cause of death due to a single infectious agent. In 2018, there were 1.5 million deaths due to TB, and 10 million new cases were reported worldwide. The major hurdle in the successful treatment of TB is the emergence of multidrug-resistant (MDR-TB) and extensively drug-resistant (XDR-TB) strains. As per the drug resistance monitoring reports, 3.4% of new cases and 18% of previously treated TB cases were MDR/rifampicin-resistant (RR)-TB cases, and 6.2% of the MDR-TB cases turned out to be XDR-TB cases [1]. Thus, there is an urgent need to discover new drugs that possess a mechanism of action different from the current drugs in TB therapy, which can effectively treat drug-resistant TB. 

Mycolic acid is a major component of the mycobacterial cell wall and is crucial for the survival of the bacteria, making it an important target in antituberculosis drug therapy [2]. Isoniazid, the first-line antituberculosis drug, inhibits mycolic acid biosynthesis. However, isoniazid is inactivated by mycobacterial arylamine *N*-acetyltransferase (NAT) [3]. NAT from *M. tuberculosis* (TBNAT) was found to be essential for intracellular survival and plays an important role in lipid metabolism [4]. During latent infection, cholesterol plays a key role in *M. tuberculosis* persistence inside macrophages. NAT is encoded in the cholesterol sterol-ring degradation gene cluster [5,6], and inhibitors explored [5,7,8] against the recombinant enzyme validated NAT as a novel therapeutic target for designing new antituberculosis drugs.

**Figure 1:**
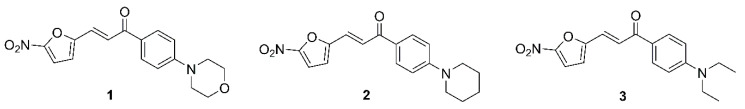
Structures of (*E*)-3-(5-nitrofuran-2-yl)-1-(4-substituted-phenyl)prop-2-en-1-one derivatives.

Nitro-containing heterocyclic compounds are promising for further investigation of their antituberculosis potential, as they are currently predominant in the TB clinical drug trial pipeline. Interestingly, the recently approved nitroheterocyclic drug, pretomanid to treat drug-resistant TB and other nitroheterocyclic clinical candidates: delamanid, nitazoxanide, macozinone (PBTZ-196) and BTZ-043 exhibit diverse mechanisms of action. Pretomanid and delamanid are mycobacterial mycolic acid biosynthesis inhibitors and are also capable of poisoning cells by generating reactive oxygen species. Nitazoxanide disrupts the membrane potential and pH homeostasis, whereas macozinone and BTZ-043 inhibit arabinogalactan synthesis by forming a covalent adduct with the decaprenylphosphoryl-ß-D-ribose-2′-epimerase (DprE1) enzyme [9]. Therefore, the exploration of diverse mechanisms of action for nitroheterocyclic compounds is justified. We have previously worked on developing 5-nitrofuran derivatives having antituberculosis potential [10]; the three most potent molecules of the series, depicted in Figure 1, based on the resazurin microtiter assay (REMA) assay results against *M. tuberculosis* H37Rv, were selected for further whole-cell phenotypic evaluation and target identification. 

## 2. Results and Discussion

The selected molecules were evaluated against *M. tuberculosis* H37Rv using an agar-based high-throughput spot culture inhibition (HT-SPOTi) assay to validate their potential to inhibit the growth of tubercle bacilli, which was also determined, independently, using a broth-culture based REMA assay [10]. The molecules were also evaluated against other bacterial strains, multidrug-resistant *M. tuberculosis*, *Mycobacterium bovis* BCG, *Mycobacterium aurum*, *Escherichia coli* and *Staphylococcus aureus*, and the respective minimum inhibitory concentration (MIC) values are provided in Table 1. Compound **2** was found to be most potent against *M. tuberculosis* H37Rv, with an MIC of 0.031 mg/L, and exhibited a lower MIC than the three first-line antituberculosis drugs (isoniazid, rifampicin and ethambutol) and pretomanid, a drug used to treat drug-resistant TB. Both compounds **1** and **2** substituted with alicyclic amines, i.e., 4-piperidino and 4-morpholino, respectively, exhibited higher activity against *M. tuberculosis* H37Rv than compound **3** with the aliphatic amino substituent, i.e., 4-diethylamino. However, compound **3** with a MIC of 0.488 mg/L against the MDR-TB strain was more potent than compounds **1**, **2** and ethambutol and equipotent to pretomanid. These compounds showed no activity against the fast-growing mycobacterium *M. aurum* up to a 500 mg/L concentration. Compounds **1** and **2** were found to be inactive against both representative Gram-negative and Gram-positive strains, *E. coli* and *S. aureus*, respectively, while compound **3** showed some activity, but it was insignificant when compared with its activity against the slow-growing mycobacterial strains. Thus, the molecules were selective towards slow-growing mycobacteria *M. tuberculosis* (drug-sensitive and multidrug-resistant) and *M. bovis* BCG. The use of broad-spectrum antimicrobial agents often attributes to the development of resistance and cross-resistance, hence, the selectivity of the compounds against mycobacteria may prove advantageous in tackling the challenge. It could be inferred that the presence of an aliphatic amine substituent, i.e., 4-diethylamino in compound **3**, made the derivatives less selective towards slow-growing mycobacteria when compared to the other alicyclic amine substituent-containing derivatives, compounds **1** and **2**. 

The cytotoxic potential of the compounds was evaluated against the two cell lines: human monocyte cell line (THP-1) and murine macrophage cell line (RAW 264.7); their respective growth inhibitory concentration (GIC) values are provided in Table 1. The compounds showed low cytotoxicity in comparison to their activities against slow-growing mycobacteria. Compounds **1** and **2** with alicyclic amine substituents showed lower cytotoxicity against both THP-1 and RAW 264.7 cell lines when compared to compound **3** with an aliphatic amine. For a better understanding of the selectivity of the molecules towards mycobacteria, the selectivity index (SI) of the compounds with respect to the slow-growing mycobacterial strains and THP-1 and RAW 264.7 cell line are shown in Table 2. The compounds exhibited excellent selectivity towards mycobacteria than the respective cell lines, with SIs ranging from 32 to as high as 16,997. The ability of the compounds to inhibit mycobacteria within macrophages was assessed using an intracellular infection model of *M. bovis* BCG infected in THP-1 macrophages. Amongst the three compounds selected, only compound **1** was found to be active at 4× MIC with an ex vivo MIC of 0.24 mg/L. The standard drug used for this study was isoniazid, which showed an ex vivo MIC of 1.25 mg/L, i.e., 2× MIC. Thus, compound **1** was found to be more potent than isoniazid in killing intracellular *M. bovis* BCG. It will be interesting to further evaluate the potential of these compounds and to measure their potency to penetrate the caseous tuberculosis lesions, which harbour the persistent bacterial subpopulation, by a caseum binding assay [11].

**Figure 2:**
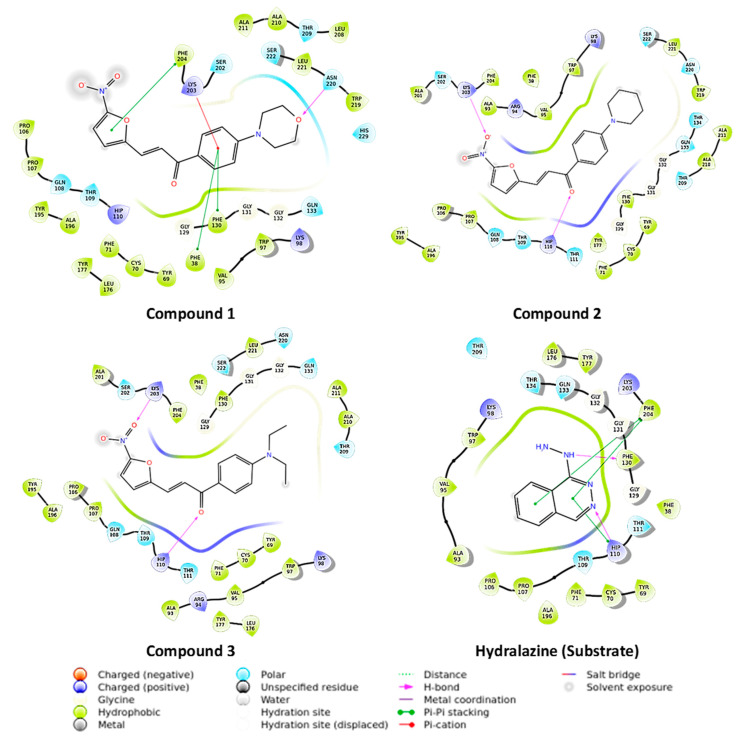
2D representation of molecules docked in *Mycobacterium tuberculosis N*-acetyltransferase (TBNAT) (PDB: 4BGF). The docking study was carried out using the Grid-based Ligand Docking with Energetics (GLIDE) module of the Maestro molecular modelling suite, Schrödinger LLC, NY.

The exploration of molecules that can inhibit novel TB drug targets is crucial for discovering agents for the effective treatment of drug-resistant TB. NAT is a novel druggable enzyme in *M. tuberculosis* involved in cell wall lipid metabolism and the detoxification of by-products of cholesterol metabolism [12]. Thus, it is a prospective drug target for developing new antituberculosis drugs. The derivatives were initially docked in *M. tuberculosis* arylamine *N*-acetyltransferase (TBNAT) (PDB: 4BGF), and then, their binding energies were assessed using molecular mechanics energies combined with generalized Born and surface area continuum solvation (MM-GBSA) to comprehend their possible interaction with the enzyme and their binding affinities. The compounds **1**, **2** and **3** exhibited extra precision GLIDE scores (XP g scores) of −3.788, −4.907 and −4.914 and MM-GBSA binding energy (dG bind) scores of −67.001 kcal/mol, −64.833 kcal/mol and −66.757 kcal/mol, respectively. The more negative XP g score and dG bind score indicate that the molecule binds better to the drug-target protein. There is no drug that is known to mechanistically inhibit NAT, hence, a comparative standard drug is not represented in Figure 2. On the other hand, the docking of the substrate hydralazine, along with the derivatives, are depicted in Figure 2. Hydralazine showed an XP g score of −6.423 and a dG bind score of −41.543 kcal/mol. The derivatives showed less negative g scores than the substrate hydralazine but showed better binding energies. The low XP g score shown by compound **1** may be attributed to a lack of interaction with His 110 (a residue of the NAT catalytic triad), whereas the high XP g score shown by hydralazine may be attributed to the two interactions it has with His 110, one hydrogen-bonding interaction and one pi-pi-stacking interaction. Thus, His 110 could be an important residue for TBNAT binding. 

The recombinant TBNAT enzyme is difficult to express in the soluble and active forms. The recombinant *Mycobacterium marinum N*-acetyltransferase (MMNAT) with 73% amino acid sequence identity is considered as the best available model for TBNAT [13,14]. Recombinant MMNAT was chosen for assessing the ability of the derivatives to inhibit mycobacterial NAT and exhibits high in vitro enzyme activity. All the amino acid residues within the binding pocket of mycobacterial NATs are either conserved or conservatively substituted [15]. Furthermore, both TBNAT and MMNAT show identical configuration of the catalytic triad (Cys70, His110 and Asp127), as reported by Abuhammad et al. [16]. The preliminary evaluation of the compounds showed that the compounds were good MMNAT inhibitors, with compound **3** being the most potent MMNAT inhibitory with a half-maximal inhibitory concentration (IC_50_) of 0.134 µM. The other two compounds **1** and **2** also showed good inhibition of MMNAT, with IC_50_ of 0.207 µM and 0.269 µM, respectively. As the compounds do not drastically differ from one another, reasons that could account for the disparity between the antimycobacterial activity and NAT enzyme inhibition need to be considered. As NAT plays a pivotal role in isoniazid metabolism/detoxification, these molecules being NAT inhibitors could also be considered hits in the development of adjunctive antituberculosis therapy to increase the efficacy of isoniazid by decreasing its metabolism by NAT. 

## 3. Materials and Methods

### 3.1. Bacterial Strains and Cell Lines

*M. tuberculosis* H37Rv (ATCC 25618), multidrug-resistant *M. tuberculosis* Peru isolate (isolate from a Peruvian patient, resistant to isoniazid and rifampicin) [17], *M. bovis* BCG (ATCC 35734), *M. aurum* (NCTC 10437), *E. coli* (NCTC 10538), *S. aureus* (ATCC 25923), human THP-1 cell line (ATCC TIB202) and murine RAW 264.7 cell line (ATCC TIB71) were used in this study. All cultures, bacterial and eukaryotic, were passaged at least twice before performing assays and were used while they were in the logarithmic phase of growth. 

### 3.2. Compounds

The derivatives evaluated in this study were chemically synthesized; their synthesis has been previously reported by us [10]. The control drugs used in this study, isoniazid, rifampicin, ethambutol, pretomanid (PA-824), ampicillin and methotrexate, were procured from Sigma Aldrich, London, UK.

### 3.3. Evaluation of Antibacterial Potential using High-throughput Spot Culture Growth Inhibition (HT-SPOTi) Assay

The compounds were analyzed using HT-SPOTi, as described previously [18]. Briefly, the compounds were dissolved in dimethyl sulfoxide, DMSO (Sigma Aldrich, London, UK) to achieve a concentration of 50 mg/mL. A range (50-0.048 mg/mL) of compound concentrations was obtained by performing a two-fold serial dilution in DMSO in sterile 96-well subskirted microtiter plates. A wider concentration range (50-0.000024 mg/mL) of the drug ampicillin was sampled against *S. aureus*. Two microliters of each dilution was added to a well in the 96-well flat-bottomed plate, to which 200 µL of agar-based media (at 65 °C) was added using a Multidrop™ Combi Reagent Dispenser (Thermo Fisher Scientific, London, UK). Middlebrook 7H10 agar (Becton Dickinson, London, UK) supplemented with 10% (v/v) oleic acid-albumin-dextrose-catalase (OADC) (Fisher Scientific, London, UK) and 0.5% (v/v) glycerol (Sigma Aldrich, London, UK) was used for mycobacterial species, whereas Luria-Bertani (LB) agar (Sigma Aldrich, London, UK) was used for *E. coli* and *S. aureus*. The plates were cooled to room temperature (RT) and 2 µL of diluted bacterial suspension, containing approximately 2 × 10^3^ bacilli, was spotted on each well. *E. coli* and *S. aureus* were incubated at 37 °C for 16 h, *M. aurum* at 35 °C for 5 days, *M. bovis* BCG and multidrug-resistant *M. tuberculosis* Peru isolate at 37 °C for 14 days. The experiments were carried out in three biological replicates (n = 3). The colonies/spot-culture growths were visually observed after the required incubation period. The lowest concentration of the compounds being tested at which no growth of bacteria was observed was reported as its MIC against the corresponding bacteria [19,20,21].

### 3.4. Cytotoxicity Assay

The cytotoxicity of the compounds against human monocyte THP-1 and murine macrophage RAW 264.7 cell lines was performed using a 96-well plate resazurin assay, as described previously [20,22]. The compounds were serially diluted (two-fold) with RPMI-1640 media (Fisher Scientific, London, UK) containing 10% fetal bovine serum (FBS) (Fisher Scientific, London, UK) in a final volume of 100 µL to achieve a concentration range of 500-0.48 μg/mL. A serial dilution to achieve a concentration range of 500-0.00024 µg/mL was carried out only for the evaluation of the cytotoxic drug, methotrexate, which was used as a positive control in the experiment. Each compound was tested in triplicate. In each well, 100 µL THP-1/RAW 264.7 cells (5 × 10^5^ cells/mL) was added, and the plates were incubated in a humidified CO_2_ incubator at 37 °C. Following a 48 h incubation period, the wells were washed twice with 1× phosphate-buffered saline (PBS) (Fisher Scientific, London, UK). As undifferentiated THP-1 cells are nonadherent, the plates were centrifuged (1000× *g*, 2 min) between each wash. Finally, 170 μL of fresh RPMI-1640 with 10% FBS was added to each well, followed by 30 µL of freshly prepared 0.01% resazurin solution (Sigma Aldrich, London, UK), and the plates were incubated overnight (16 h) under the conditions mentioned above. The experiments were carried out in three biological replicates (n = 3). At the end of the incubation period, the color of the media in each well was observed. A color change from blue to pink indicated active respiratory processes in live cells. The lowest concentration of the compound at which no viable eukaryotic cells were detected (blue media) was reported as the GIC.

### 3.5. Intracellular Survival Assay

The intracellular survival of mycobacteria was assessed using the *M. bovis* BCG infected in THP-1 macrophages model. The THP-1 monocyte cells (2 × 10^5^ cells/well) were differentiated into macrophages using 100 nM phorbol 12-myristate 13-acetate (PMA) (Sigma Aldrich, London, UK) in RPMI-1640 (Fisher Scientific, London, UK) containing 10% fetal bovine serum (FBS) (Fisher Scientific, London, UK) in 24-well plates and incubated at 37 °C, 5% CO_2_ for 48 h. After the 48 h incubation, the media was aspirated out of the well, and the macrophages were washed twice with RPMI-1640 containing 10% FBS. The macrophages were infected with *M. bovis* BCG to achieve a multiplicity of infection (MOI) of 10:1 and were incubated at 37°C, 5% CO_2_ for 3 h. After the infection stage, the culture was washed with RPMI-1640 twice and incubated for 48 h with different concentrations of the compounds (4×, 2×, 1×, 0.5× and 0.25× MIC) in RPMI-1640 media containing 10% FBS. Following the incubation, the cells were washed twice with RPMI-1640 and lysed in 500 μL of sterile distilled water at room temperature (RT) for 10 min. The lysed cells were centrifuged 16,000× *g* for 10 min at RT. They were resuspended into 50 μL of sterile distilled water, and then, 5 μL was spotted onto the 24-well plates containing Middlebrook 7H10 agar containing 10% (*v/v*) of OADC supplement. The plates were incubated at 37 °C for 14 days, following which, they were checked for the growth of bacterial colonies to determine intracellular survival. The experiment was carried out in three biological replicates (n = 3) [23,24]. 

### 3.6. Molecular Docking and MM-GBSA of the Molecules

Docking studies were carried out using a Grid-based Ligand Docking with Energetics (GLIDE) module of Maestro molecular modelling suite, Schrödinger LLC, NY. The protein structure PDB code: 4BGF for TBNAT was retrieved from Protein Data Bank (www.rcsb.com) and was subjected to protein preparation. The protein was energy-minimized until the root mean square deviation (RMSD) of 0.3 Å using the imperf tool of the protein preparation wizard in Maestro, and the fully prepared protein was analyzed using a Ramachandran plot. The receptor grid was generated using the centroid of the Phe 130 residue, as the PDB of the protein did not contain a co-crystalized ligand. The ligands were prepared using Ligprep, where appropriate hydrogens were added to all structures, and subsequently, energy minimization was carried out using the OPLS-2005 force field with a constant dielectric of 1.0 [25]. The ligands were docked using the extra-precision (XP) mode of the GLIDE module. The more negative the XP g score, the better is the docking of the molecule in the receptor protein [26].

MM-GBSA estimates the free energy of the binding of small ligands to the biological macromolecules. The more negative the dG bind score, which is expressed in kcal/mol, the better is the ligand-binding affinity with the receptor. A constraint of 5 Å flexibility of the protein around the ligand was applied for the study [27]. 

### 3.7. Estimation of Inhibition against Recombinant Mycobacterium Marinum N-acetyl Transferase (MMNAT) 

MMNAT was expressed in *E. coli* BL21(DE3)pLysS cells transformed with the pET28b(+) vector containing the *nat* gene (MMAR_5055) insert and purified using nickel (Ni^2+^) ion affinity chromatography, as described previously [13,28]. Ten microliters of MMNAT enzyme (0.1 µg/µL), i.e., 1 µg of the enzyme in each well, was incubated with 45 µL of hydralazine (Sigma Aldrich, London, UK) (final concentration: 450 µM) for 5 min at RT. One microliter of DMSO solution containing the compound was added to achieve the appropriate concentration. Forty microliters of acetyl CoA (Sigma Aldrich, London, UK) (final concentration: 400 µM) was added to each well to start the reaction. The reaction mixture was incubated for 20 min at RT. Twenty-five microliters of stop solution, i.e., 5 mM 5,5-dithio-bis-(2-nitrobenzoic acid) (DTNB) (Sigma Aldrich, London, UK) solution in 6.4 M guanidine HCl (Sigma Aldrich, London, UK) (pH 8), was added to all the wells. The plates were read at 405 nm. The experiment was carried out in two biological replicates (n = 2). There were two controls set up: one with no enzyme (0% enzyme activity) and one with no inhibitor (100% enzyme activity). Percentage of inhibition was calculated using a negative control (0% enzyme activity) and enzyme reaction (100% enzyme activity), which were further used to generate a linear regression from which IC_50_ of the compounds was calculated through extrapolation (Supplementary Materials) [7,29].

## 4. Conclusions

This study identified highly potent, selective and mycobactericidal hits with a novel mechanism of action. Compound **2** was found to be more potent than the three first-line TB drugs (isoniazid, rifampicin and ethambutol) and pretomanid against *M. tuberculosis* H37Rv. Compound **3** exhibited a higher activity than ethambutol and was equipotent to pretomanid against MDR-TB. The compounds exhibited a bactericidal mode of action when evaluated against *M. bovis* BCG. Compound **1** was effective in inhibiting *M. bovis* BCG within THP-1 macrophages and could be considered for evaluation in a dormant TB model. The compounds showed selectivity towards *M. tuberculosis* H37Rv, MDR-TB and *M. bovis* BCG when compared to Gram-positive and Gram-negative bacteria. The compounds showed low cytotoxicity and excellent selectivity towards mycobacteria when compared to THP-1 and RAW 264.7 cell lines. We, for the first time, have identified 5-nitrofuran as a prospective scaffold for mycobacterial NAT inhibition, and it could be used as a basis to design mycobacterial NAT inhibitors in the future. The compounds being potent and selective antimycobacterial agents could be further studied for their prospects of being developed into preclinical/clinical candidate(s).

## Figures and Tables

**Table 1:** Minimum inhibitory concentrations (MICs) of the derivatives and drug standards against different species of bacteria, growth inhibitory concentrations (GICs) for cell lines and half-maximal inhibitory concentration (IC_50_) for MMNAT.

Compound	MIC (mg/L)						GIC (mg/L)		IC_50_ (µM) (mg/L)
	Mtb H37Rv	MDR-TB	*Mycobacterium bovis* BCG	*Mycobacterium aurum*	*Escherichia coli*	*Staphylococcus aureus*	THP-1 Cell Line	RAW 264.7 Cell Line	MMNAT
**1**	0.244	0.976	0.06	NA^a^	NA^a^	NA^a^	62.5	125	0.207 (0.068)
**2**	0.031	1.953	0.0075	NA^a^	NA^a^	NA^a^	62.5	125	0.269 (0.088)
**3**	0.488	0.488	0.03	NA^a^	250	62.5	15.6	31.25	0.134 (0.042)
**Isoniazid**	0.80	NA^a^	0.625	1.25	ND	ND	NA^a^	NA^a^	NA^b^
**Rifampicin**	0.125	NA^a^	0.50 ^#^	0.40 ^#^	ND	ND	ND	ND	NA^b^
**Ethambutol**	2.00	8.00	2.00 ^#^	0.10 ^#^	ND	ND	ND	ND	NA^b^
**Pretomanid**	0.061	0.488	ND	ND	ND	ND	ND	ND	NA^b^
**Ampicillin**	ND	ND	NA^a^	ND	2.00	0.125	ND	ND	ND
**Methotrexate**	NA^a^	250	500	ND	ND	ND	0.975	0.122	ND

Mtb: *Mycobacterium tuberculosis*, MDR-TB: multidrug-resistant *M. tuberculosis*, ND: not determined, NA^a^: not active until 500 mg/L concentration, NA^b^: does not exhibit 50% inhibition, i.e., IC_50_ against *M. marinum* arylamine *N*-acetyltransferase (MMNAT) up to 1 µM concentration and ^#^ MICs from reference Gupta and Bhakta, 2012.

**Table 2:** Selectivity indices of the derivatives (cell line vs. mycobacteria).

Compound	SI					
	THP-1			RAW 264.7		
	Mtb H37Rv	MDR-TB	*M. bovis* BCG	Mtb H37Rv	MDR-TB	*M. bovis* BCG
**1**	256	64	1042	512	128	2083
**2**	2016	32	8333	4032	64	16997
**3**	32	32	520	64	64	1042

Mtb: *M. tuberculosis,* MDR-TB: multidrug-resistant *M. tuberculosis*. The selectivity index (SI) was calculated by dividing the GIC for the cell line by the MIC for the bacteria; the SI was not calculated with respect to *M. aurum*, *E. coli* and *S. aureus*, as the compounds are inactive or showed low activity against the same.

## Supplementary Materials

The following are available online at https://www.mdpi.com/2079-6382/9/7/368/s1. Figure S1: Enzyme inhibition plots of compounds 1, 2 and 3 against *M. marinum* NAT.
