# Supplementary material for: 3-(5-Nitrofuran-2-yl)prop-2-en-1-one Derivatives, with Potent Antituberculosis Activity, Inhibit A Novel Therapeutic Target, Arylamine N-acetyltransferase, in Mycobacteria
Source: Antibiotics (Basel). 2020 Jul 1;9(7):368. doi: 10.3390/antibiotics9070368 (PMC7400135; doi:10.3390/antibiotics9070368)
Supplement: Supplementary file 1 [file antibiotics-09-00368-s001.zip › antibiotics-816058-supplementary.docx]

Supplementary Figure 1: Enzyme inhibition plots of compounds 1, 2 and 3 against *M. marinum* NAT. The IC50 were calculated as an extrapolation of the linear regressions obtained. The experiments were performed with two biological replicates containing triplicates within each experiment.
